# Supplementary material for: On China’s image constructed from western news coverage of China’s humanitarian aid
Source: PLoS One. 2025 Jun 18;20(6):e0326214. doi: 10.1371/journal.pone.0326214 (PMC12176173; doi:10.1371/journal.pone.0326214)
Supplement: S2 — (DOCX) [file pone.0326214.s002.docx]

# S2 File. Coding Scheme for Metaphor Identification and Categorization

This coding scheme outlines the procedure used to identify and classify metaphorical expressions in the Sub-corpus CHAE. The analysis draws on the Metaphor Identification Procedure (MIP) and its revised version (MIPVU), as well as conceptual structures developed under the framework of Conceptual Metaphor Theory (CMT).

## 1. Identification of Metaphorical Expressions

Following the MIP/MIPVU approach, each lexical unit in the text was evaluated in context to determine whether its meaning contrasts with a more basic contemporary meaning, and whether it could be understood via comparison. Expressions meeting these criteria were marked as metaphorical.

## 2. Categorization by Conceptual Metaphor

Identified metaphorical expressions were categorized under the overarching conceptual metaphor THE NATION AS PERSON. This included subcategories such as CHINA AS POLITICIAN, CHINA AS SALESMAN, CHINA AS AGGRESSOR, and CHINA AS DICTATOR.

## 3. Sample Classification Table

| Expression | Metaphor Type | Interpretation |
| --- | --- | --- |
| China’s so-called aid | CHINA AS POLITICIAN | Skepticism of China’s intentions |
| Chinese largesse; the fastest turnaround in global history from pariah to hero | CHINA AS UPSTART | Implied skepticism |
| were purchased, not donated, were found to be defective | CHINA AS VENDOR | Portrays China as forceful and dominant |
| An insolent dictator | CHINA AS DICTATOR | Emphasizes authoritarian behavior |
