# Supplementary material for: On China’s image constructed from western news coverage of China’s humanitarian aid
Source: PLoS One. 2025 Jun 18;20(6):e0326214. doi: 10.1371/journal.pone.0326214 (PMC12176173; doi:10.1371/journal.pone.0326214)
Supplement: S4 — (DOCX) [file pone.0326214.s004.docx]

# S5 File. Example Quotes for Metaphorical Expressions

This file includes key metaphorical expressions cited in the qualitative analysis. Each quote is presented with the metaphor it exemplifies, the media source, and the interpretation context.

## Metaphor Type: CHINA AS POLITICIAN

But Chinas mask diplomacy has fanned a mistrust of the EU kindled years ago by Italy’s populists. The legislator sounded despondent.“I wish I could say this mask diplomacy isn’t working very well. But it is, unfortunately.”(*The Guardian*, 2020-04-29)

Relations with Europe also have taken a turn for the worse, precipitated by China’s ill-conceived attempts to score a propaganda win by supplying poor quality face masks and other medical equipment to countries afflicted by the coronavirus. Chinese officials compounded the fiasco of their mask diplomacy by faulting Europeans for their allegedly poor crisis management. (*The Australian*, 2020-06-16)

The campaign was not all punitive, though; it also included incentives for good behavior. One facet of the response was “mask diplomacy”: wielding China’s near-monopoly over essential P.P.E. manufacturing as a tool for rewarding friends and punishing perceived enemies. (*The New York Times*, 2021-7-11)

Interpretation: Describes China's diplomatic aid as calculated and conditional.

## Metaphor Type: CHINA AS DICTATOR

China is also a systemic rival, however, and it is increasingly going on the offensive, also vis-a-vis Europe. Beijing’s “mask diplomacy” coupled with a disinformation campaign in the midst of the coronavirus crisis is just one current example. The leadership of the authoritarian, one-party state passes up no opportunity to drive a wedge between the EU member states and weaken them. We are locked in a tough competition of values stemming from very different concepts of society. (*Der Spiegel*，2020-08-02)

Interpretation: Frames China's assistance as authoritarian influence.

## Metaphor Type: CHINA AS SALESMAN

European frustrations with Chinese policies have been mounting, but they crystallized this year in the wake of the coronavirus pandemic. China’s obfuscation of its early missteps in containing the coronavirus and its failure at “mask diplomacy” soured public sentiment in several countries, especially the Netherlands and Spain, where protective gear and other supplies that were purchased, not donated, were found to be defective. (*The New York Times*,2020-09-22)

Interpretation: Suggests a commercialized and image-oriented aid effort.

## Metaphor Type: CHINA AS AGGRESSOR

This spring, Beijing energetically promoted its exports and overseas donations of medical supplies and asked foreign politicians to thank China publicly for the shipments. …The study, however, cast doubt on whether the humanitarian aid blitz really took place, since China’s exports were down in March from a year earlier. (*The New York Times*，2020-05-06)

Interpretation: Conveys grudging acknowledgment of China’s strategic success with negative undertone.
