# Supplementary material for: On China’s image constructed from western news coverage of China’s humanitarian aid
Source: PLoS One. 2025 Jun 18;20(6):e0326214. doi: 10.1371/journal.pone.0326214 (PMC12176173; doi:10.1371/journal.pone.0326214)
Supplement: Attachment — Submitted filename: reponse to reviewer.pdf [file pone.0326214.s008.pdf]

Dear Editor Rafael Galvão de Almeida,

Thank you very much with your decision letter along with the review of my paper（manuscript # PONE-D-24-60013）. I feel encouraged by your comment “it has merit” with a minor revision. I’d like to show my gratitude for the profound comment. Therefore, I plan to revise my paper now meticulously. As a result of the extremely substantial revisions I made, the current version of the paper is the revised manuscript corresponding to the Reviewer’s comment point to point. Below I describe in detail how I addressed the issues that the reviewer raised and what changed I made.

Besides the modifications I’ve made following the Reviewer’s suggestion, I have also carefully revised the sub-section headings to more accurately reflect the empirical content presented, so as to keep the academic rigorous standard. The previous sub-heading “4.1 Reporting Timeline” has been revised to “4.1 Reporting Distribution Over Time”, in order to better describe the changes in reporting intensity throughout the observed period. The previous sub-heading “4.2 The Narrative Behind ‘Mask Diplomacy’” has been revised to “4.2 Frequency and Modifier Analysis of ‘Mask Diplomacy’”, to more precisely capture the focus on lexical patterns and the semantic framing strategies employed by the media. These adjustments aim to enhance the structural transparency and facilitate the readers’ understanding of the empirical results presented.

Followings are my point-to-point reponse to Reviewer#1:

1. Methodology section

a. I noticed that in your manuscript, Section 3.2 is titled “Methodology.” However, “methodology” generally refers to a broader framework that encompasses the overall approach, including data, tools, and analysis. In Section 3.2, you only describe the specific method used in this project. Therefore, I recommend renaming Section 3 as “Methodology” and Section 3.2 as “Method.”

Response: Thank Reviewer#1 for pointing this out. This was indeed an oversight on my part during the previous revision process. As you rightly noted, Section 3 should be titled **“Methodology”** to reflect the overall research framework, while Section 3.2 should be labeled **“Method”** to describe the specific procedures used. I have now corrected the section headings accordingly. I appreciate your careful attention to this detail—it has helped improve the manuscript’s clarity and precision. Section 3 is now titled **“Methodology”** to reflect the overall research design, including corpus construction, analytical tools, and theoretical orientation. Section 3.2 has been renamed **“Methods”**, focusing specifically on the procedures for identifying and analyzing conceptual metaphors.

b. Additionally, the second paragraph of Section 3.1.1 contains extensive explanations about the criteria for selecting the newspapers. To enhance clarity and conciseness, you might consider presenting that information in a table.

Response: Thank Reviewer#1 for this helpful suggestion. I agree that presenting the selection criteria and background of the newspapers in tabular form would improve both clarity and readability. I have now reorganized this information into a new table in Section 3.1, which summarizes each outlet's geographic location, political stance, ownership structure, and rationale for inclusion in the corpus.

c. I also suggest combining Sections 3.1.1 (Data Collection) and 3.1.2 (Corpus Construction) since they are closely related and share overlapping content.

Response: Thank Reviewer#1 for the suggestion. I fully understand the rationale for proposing a streamlined structure. However, based on earlier reviewer feedback from a previous round, I was advised to elaborate more clearly and separately on the data collection process and corpus construction procedures, as the earlier description was considered too concise, as Reviwer #1 advised to "Provide clearer explanation of corpus design, including selection criteria and data composition.".

Furthermore, given that two distinct corpora were developed for this study (Corpus CC and Sub-corpus CHAE), I believe maintaining Sections 3.1.1 and 3.1.2 as separate but logically connected units better reflects the complexity of the research design and improves the manuscript's clarity. Therefore, I have chosen to retain the current organization while ensuring that the two sections are closely related and avoid redundancy. I sincerely appreciate his/her suggestion, which prompted me to refine the transitions between the two subsections for better coherence.

d. Moreover, some details within the current methodology section appear to be better suited for the "Findings" section. Please consider relocating this content accordingly.

Response: Thank Reviewer#1 for pointing this out. Upon review, I agree that certain descriptive elements—particularly those involving preliminary observations and early data patterns—are more appropriate for the **Findings** section. I have revised the Methododology section to ensure that it focuses solely on describing the data sources, corpus construction, and analytic procedures. Content that involved preliminary interpretation of findings, such as the identification of THE NATION AS PERSON and its secondary mappings (e.g., CHINA AS PERSON, EUROPE AS PERSON), has been relocated to the Findings section. Now, the Methodology section exclusively outlines the procedures without reporting research outcomes, in line with academic writing conventions. I sincerely appreciate his/her suggestion to improve the academic rigor of the manuscript.

e. Finally, the manuscript would benefit from a dedicated data analysis section that demonstrates, for example, showing your readers an example about how metaphor(s) is identified in the dataset (i.e., MIP or MIPVU).

Response: Thank Reviewer#1 for this constructive suggestion. I fully agree that providing a concrete example of the metaphor identification process enhances the transparency and replicability of the study. In the revised manuscript, I have, as the reviewer suggested, added a dedicated **Data Analysis** section (Section 3.3) that systematically describes the procedure based on the MIPVU guidelines. Moreover, I included a detailed example from *The Guardian* (April 29, 2020), where the expression "fan mistrust" was analyzed to illustrate how contextual and basic meanings were compared to determine the metaphorical status. I believe this addition strengthens the methodological rigor and clarity of the paper.

f. Based on these points, I recommend restructuring the methodology section as follows:
   3. Methodology
   3.1 Data
   3.2 Methods
   3.3 Data Analysis
   I hope these suggestions help improve the clarity and structure of your manuscript.

Response: Thank Reviewer#1 very much for your rigorous and constructive feedback. I do benefit tremendously with the recommendation of structuing the methodology section. Hence, I have revised the structure of the Methodology section accordingly. The section is now organized into three subsections: 3.1 Data, 3.2 Methods, and 3.3 Data Analysis. In addition, I have expanded subsection 3.3 to explicitly describe the basic corpus statistics and the metaphor identification procedures, thereby enhancing the transparency and clarity of the methodological design. I sincerely appreciate his/her valuable suggestions, which have helped to improve the overall coherence and academic rigor of the manuscript.

2. Terminology issues:

a. AntConc
   In the quantitative analysis section, you refer to AntConc 3.5.9 as **a vocabulary analysis software**. However, AntConc is more described as a "corpus analysis toolkit" (please also cite from Anthony). I recommend updating the description to reflect its true functionality.

Response: Thank Reviewer for highlighting this important terminology issue. I have revised the description of AntConc in the Methods section to accurately refer to it as a **"corpus analysis toolkit"**, following the terminology used by Anthony (2023). The relevant citation has also been added to properly acknowledge the source. Thanks again for his/her valuable and meticulous correction, which has helped me improve the academic accuracy of my manuscript.

b. MIP vs. MIPVU

In Section 3.2, the manuscript states that the MIP method was utilized in this project, while in Section 4.4, a different method, MIPVU, is mentioned. Please ensure consistency in the naming of the method throughout the manuscript and cite the related reference for the chosen method.

Response: Thank Reviewer for drawing my attention to this inconsistency. I have carefully reviewed the manuscript and corrected all mentions to consistently refer to the MIPVU method throughout the text. In this study, the Metaphor Identification Procedure Vrije Universiteit (MIPVU) (Steen et al., 2010) was adopted to identify metaphorical expressions within the corpus. Compared to the original Metaphor Identification Procedure (MIP) proposed by the Pragglejaz Group (2007), MIPVU offers a more detailed and standardized framework for linguistic metaphor identification, particularly suitable for large and complex datasets such as media discourse. Its explicit coding conventions and expanded categorization criteria enhance the transparency, consistency, and replicability of metaphor analysis, aligning with the requirements of corpus-based critical discourse studies.

Additionally, I have included the appropriate citation for MIPVU, following Steen et al. (2010), in both the Methods and Data Analysis sections, and added the corresponding reference to the References list. Thanks again for his/her valuable feedback, which has helped me improve the accuracy terminology in my manuscript.

c. Section 4 (Results)
   Please note the essential difference between "Results" and "Findings":
   The former one should present the empirically obtained data, including statistical analyses, charts, and other measurable experimental indicators, whereas the later one is intended for interpreting, summarizing, and analyzing these data.

Response: Thank Reviewer for his/her thoughtful comment regarding the distinction between "Results" and "Findings". I appreciate his/her academic explanation. In response, I have made the following revisions. Upon reflections, I recognized that parts of the section not only presented empirical data but also included preliminary analysis and summarization of the findings based on the data. Therefore, to more accurately reflect the content and maintain terminological precision, following the Reviewer's suggetion, I have revised the section title to "Findings" in the updated manuscript. I hope this adjustment clarifies the structure and better to follow academic conventions.

Followings are my point-to-point reponse to Reviewer#2:

Reviewer #2: The research question is not articulated logically enough. The current introduction does not adequately establish clear theoretical or empirical gaps, making it difficult

to logically derive the research questions posed. I would suggest that the authors further elaborate on the limitations of previous research or highlight areas that have not been adequately explored in the literature, thus providing a more coherent theoretical rationale for the study. This would help position the research question more convincingly within the existing academic context.

Response: Thank Reviewer #2 for this valuable suggestion. I agree that the original introduction could be improved in its articulation of the theoretical and empirical gaps. In the revised manuscript, I have thoroughly revised the **Introduction** and **Literature Review** sections to address this issue. Specifically, I now clearly outline three underexplored aspects in the existing literature: (1) the lack of corpus-based analyses of Western media coverage of China's humanitarian aid during COVID-19; (2) the absence of research combining both quantitative corpus techniques and qualitative metaphor analysis; and (3) the under-examined ideological implications of the label "mask diplomacy." These gaps are now explicitly presented in **Section 1, final paragraph (pp. 2–3)**, leading logically to the two research questions. I hope this revision provides a more coherent and convincing rationale for the study.

Some parts of the discussion lacked adequate support from the literature. Whilst the interpretation of the findings makes some sense, certain points are made without sufficient citation of relevant research. I would recommend that the authors cite more of the existing literature in their discussion, especially regarding media-specific points. This would enhance the academic rigour and credibility of the discussion.

Response: I sincerely thank the reviewer#2 for this insightful and constructive suggestion. In response, I undertook a substantial revision of the **Discussion section** to address the issue of insufficient literature support and to improve overall conceptual clarity and depth.

Firstly, I have now restructured the main discussion part into three distinct subsections (5.1, 5.2, 5.3 and 5.4) in order to better differentiate between lexical strategies, metaphorical construction, media-specific ideological framing, and different media positioning. This restructuring not only improves the clarity and logical flow but also enables us to better align each interpretive dimension with appropriate theoretical and empirical literature.

Originally, the discussion consisted of: 5.1. China's image in western media's discursive strategies in THE NATION AS PERSON; 5.2. Ideological implication of THE NATION AS PERSON;5.3. Media outlet's positionality and its framing of nation's image. However, I found that the original Section 5.1 was internally heterogeneous, discussing both the discursive use of "mask diplomacy" and the metaphorical construction of China's image under the "nation-as-

person” schema. This not only blurred the topical focus of that section but also led to significant conceptual overlap with Section 5.2.

Secondly, I have significantly strengthened the discussion section by integrating a wider range of relevant academic literature to better support our interpretations and clarify the theoretical positioning of our findings. The following modifications have been made to improve academic rigour:

5.1. Lexical Framing of “Mask Diplomacy”

- Strengthened theoretical grounding by referencing Lakoff & Johnson’s (1980) Conceptual Metaphor Theory and Charteris-Black’s (2004) Critical Metaphor Analysis.
- Responded to prior research on “mask diplomacy”, including Kowalski (2021), Fahrisa et al. (2023), and Müller et al. (2024). I clarified that while these studies emphasize geopolitical and diplomatic interpretations, my contribution lies in examining how the term is linguistically operationalized in Western news discourse.
- Contribution clarified: I positioned my study as an extension of these works, offering a corpus-based discourse-linguistic perspective that highlights how evaluative language and metaphorical choices function ideologically.

5.2. China’s image in western media’s discursive strategies in THE NATION AS PERSON

- Broadened literature context by incorporating work on metaphor in media discourse (e.g., Charteris-Black, 2004; Zhang & Lin, 2023).
- Clarified thematic novelty: I explained that while many metaphor studies focus on war, journey, or spatial metaphors, our study foregrounds personification metaphors such as “CHINA AS POLITICIAN” and “AMERICA AS HOTHEAD.”

5.3. Media Outlet’s Positionality and Framing

- Supported claims with empirical literature, including studies on media ownership, ideology, and geopolitical bias [10], [12], [21], [25], [30], [35], [38].
- Highlighted contribution: I explained how my comparative analysis of The New York Times, The Guardian, and The Australian provides a fine-grained account of metaphor variation across outlets—showing how the same metaphor (e.g., CHINA AS POLITICIAN) is differently realized depending on political stance, ownership, and target readership.

In summary, I have expanded the discussion from two to three structured subsections; enhanced each part with relevant theoretical and empirical citations;clarified how this study extends and enriches prior research; improved the overall academic rigour and interpretive precision of the

manuscript.

I am grateful for the reviewer#2 ’s valuable input, which has significantly improved the clarity, coherence, and scholarly depth of the revised manuscript.

Thank you very much again for these meticulous suggestions. I do benefit a lot and revised mostly to resubmit my paper.

Sincerely,

Lei Sun

Tianjin Normal University
