# Supplementary material for: On China’s image constructed from western news coverage of China’s humanitarian aid
Source: PLoS One. 2025 Jun 18;20(6):e0326214. doi: 10.1371/journal.pone.0326214 (PMC12176173; doi:10.1371/journal.pone.0326214)
Supplement: Attachment — Submitted filename: comments.docx [file pone.0326214.s005.docx]

1. Please maintain consistency in terminology usage, particularly regarding capitalization - e.g., covid-19, COVID-19, and China's Medical Assistance. When a convention is established, it should be maintained throughout the manuscript.
2. Please review and ensure full compliance with APA formatting guidelines.
3. The connection between the literature review and your research findings requires strengthening (notably, the discussion section lacks citations). Specifically, the rationale for employing Critical Discourse Analysis (CDA) needs clarification. If CDA is being utilized as a theoretical framework, it should be thoroughly introduced in the literature review, potentially in section 2.2. Consider incorporating the following references to enhance theoretical depth:
a. Lu, G., & Yu, Y. (2024). The use of metaphors to construct crisis discourses in describing COVID-19 vaccines in the Chinese and the American news media: A corpus-assisted critical approach. *Journal of Language and Politics*, 23(4), 520-543.
b. Yu, Y., Lu, G., Fung Sin, K., & Yu, Z. (2024). Media representations of China’s inclusive education: A corpus-assisted critical discourse analysis. *PLoS ONE*, 19(3), e0300123.
c. Lu, G., Chen, J., & Zhang, L. (2022). The image construction of the City Yiwu in overseas media: Corpus-driven research based on 2010-2015 and 2016-2021. *Linguistics and Culture Review*, 6(1), 162-176.
4. The data selection methodology requires restructuring. Several points need addressing:
a. Please justify why secondary searches using "China's emergency medical" were conducted in WENRCC rather than directly in Factiva
b. The corpus size (22 articles) appears insufficient for robust analysis (specific token counts needed)
c. The frequency tables showing predominantly single occurrences raise reliability concerns
d. CDA typically acknowledges ideological variations even within national media (broadsheets, tabloids, left-wing, right-wing outlets). The rationale for aggregating news coverage from different countries requires further justification, particularly regarding shared ideological perspectives
5. Sections 4.1-4.2 could benefit from implementing a keyword list-driven framing theory approach. Consider referencing:
a. Ming, L., & Ma, J. (2022). The politics of fear in Hong Kong protest representations: A corpus-assisted discourse study. *Journal of Language and Politics*, 21(1), 37-59.
b. Liu, M., & Huang, J. (2024). Framing responsibilities for climate change in Chinese and American newspapers: A corpus-assisted discourse study. *Journalism*, 25(8), 1792-1811.
6. The Discussion section requires substantial revision to address the aforementioned concerns.
