# Supplementary material for: On China’s image constructed from western news coverage of China’s humanitarian aid
Source: PLoS One. 2025 Jun 18;20(6):e0326214. doi: 10.1371/journal.pone.0326214 (PMC12176173; doi:10.1371/journal.pone.0326214)
Supplement: Attachment — Submitted filename: Response to Reviewers.docx [file pone.0326214.s006.docx]

Dear Editor Rafael Galvão de Almeida,

Thank you very much with your decision letter along with the two reviews of my paper（manuscript # PONE-D-24-60013）. I’ve read your letter and reviews very meticulously. I’d like to show my gratitude for the two profound comments. I understand that I need to make very substantial revisions and I’ve been doing it for a month. I believe I will be able to successfully make all the revisions that the two reviewers suggested because the suggested/requested revisions are all quite specific and detailed even though they will involve a lot of work. An equally important motivation for me to revise the paper is that all two reviewers were very encouraging, as Review #2 commented “The manuscript is technically sound, and the data supports the conclusions” and “The statistical analysis has been performed appropriately and rigorously”. Both the two reviewers agreed “the author has made all data underlying the findings in their manuscript fully available” and “the manuscript presented in an intelligible fashion and written in standard English”. I have been encouraged and inspired by these two reviewers’ comments.

Therefore, I plan to revise my significantly revised paper now. As a result of the extremely substantial revisions I made, the current version of the paper is essentially a new manuscript except that the data and results of the study remain the same. Below I describe in detail how I addressed the issues that you and the reviewers raised the what changed I made. My description of the changes and responses I made in the revision is organized into two main parts. 1)changes/responses addressing the academic editor’s requests and issues and 2)changes/responses addressing the specific comments/suggestions made by each reviewer.

**1)Changes/responses addressing the academic editor’s requests and issues**

1.Please ensure that your manuscript meets PLOS ONE’s style requirements, including those for file naming.

**Response:** Thank Editor Rafael Galvão de Almeida for highlighting the importance of adhering to PLOS ONE’s style and formatting requirements. I have revised the manuscript to fully comply with these guidelines, including proper file naming conventions, manuscript structure, and formatting of titles, authors, affiliations, abstract, headings, and references.

2. Thank you for stating the following financial disclosure:

“Tianjin Education Commission, China.”

Please state what role the funders took in the study.  If the funders had no role, please state: "The funders had no role in study design, data collection and analysis, decision to publish, or preparation of the manuscript."

If this statement is not correct you must amend it as needed.

Please include this amended Role of Funder statement in your cover letter; we will change the online submission form on your behalf.

**Response:** Thank Editor Rafael Galvão de Almeida for his reminder regarding the clarification of the funder’s role. I confirm that the funding agency, Tianjin Education Commission, China, had no involvement in this research. Therefore, I have included this amended “Role of Funder” statement in the cover letter “**The funders had no role in study design, data collection and analysis, decision to publish, or preparation of the manuscript.”**

3. Thank you for stating the following in the Acknowledgments Section of your manuscript:

“This work was supported by Tianjin Education Commission Research Project, China [grant number 2024SK022];”

We note that you have provided funding information that is not currently declared in your Funding Statement. However, funding information should not appear in the Acknowledgments section or other areas of your manuscript. We will only publish funding information present in the Funding Statement section of the online submission form.

Please remove any funding-related text from the manuscript and let us know how you would like to update your Funding Statement. Currently, your Funding Statement reads as follows:

“Tianjin Education Commission, China.”

Please include your amended statements within your cover letter; we will change the online submission form on your behalf.

**Response:** Thank Editor Rafael Galvão de Almeida for pointing out this issue regarding the placement of funding information. In response, I have made the following revisions.I have removed the funding-related text from the Acknowledgments section, as instructed.

Also, I have updated Funding Statement in the Cover Letter: “This work was supported by Tianjin Education Commission Research Project, China [grant number 2024SK022]. The funders had no role in study design, data collection and analysis, decision to publish, or preparation of the manuscript.” Thanks again for your careful review.

**2)changes/responses addressing the specific comments/suggestions made by each reviewer.**

**REVIEWER #1**

1.Ensure consistent formatting throughout the manuscript according to the journal guidelines.

**Response:** Thank Review #1 for his/her valuable feedback. I have thoroughly reviewed my manuscript and revised the formatting to ensure consistency throughout, strictly following the PLOS ONE journal guidelines. Specifically, I have standardized the formatting of headings and subheadings. I have ensured that in-text citations and reference lists adhere to the journal’s citation style. I have adjusted the formatting of figures, tables, and their legends. I have uniformly applied the required font styles, sizes, and line spacing across the manuscript.

I believe these revisions enhance the overall clarity and presentation of my manuscript. Please refer to the revised version for confirmation of these changes.

2. Provide clearer explanation of corpus design, including selection criteria and data composition.

**Response:** Thank Review #1 for his/her insightful comment regarding the corpus design. In response, I have revised the manuscript to provide a clearer and more detailed explanation of my corpus design, including the selection criteria and data composition. Specifically, I have added additional details in the Methods section to clarify the following:

**a. Data Sources and Retrieval Process**: I now explain that my corpus was initially constructed using the Factiva database, with articles retrieved using keywords such as “COVID-19,” “China,” and related terms.

**b. Inclusion and Exclusion Criteria**: I have specified the criteria used for selecting articles, including language (English), publication from reputable Western news outlets, and relevance to the COVID-19 context and China’s role.

**c. Corpus Composition**: The manuscript now details the composition of my corpus. For example, my primary corpus (CC) comprises 9,783 articles retrieved between December 30, 2019, and June 1, 2024, which was manually screened to yield 9,251 valid articles. A sub-corpus (CHAE) was then created by further filtering these articles using specific keywords (e.g., “China’s emergency humanitarian aid” and “mask diplomacy”), resulting in 22 initial samples and 21 validated articles.

**d. Token Counts and Data Scope**: I have included token count details and further information on the scope of the data to demonstrate the robustness of our dataset.

**e. Rationale for Secondary Searches**: Finally, I clarify why secondary searches (using “China’s humanitarian aid”) were conducted within the refined corpus (CC) rather than directly in Factiva, emphasizing the enhanced relevance and specificity of the data.

I believe that these revisions have improved the clarity and transparency of my corpus design and methodology. Thank Review #1 for helping me enhance my manuscript.

3. Strengthen connections between literature review and discussion sections, with appropriate citations.

**Response:** Thank Review #1 for highlighting the importance of strengthening connections between the literature review and discussion sections. In response to his/her suggestion, I have carefully revised the manuscript to explicitly link key themes and findings in the Discussion back to relevant scholarship reviewed in the Literature Review, incorporating appropriate citations throughout. Specifically, I have made the following modifications:

**a. Clarification and Expansion of CDA in the Literature Review** : I have explicitly introduced Critical Discourse Analysis (CDA) as a foundational theoretical framework, clearly explaining its relevance and methodological alignment with Critical Metaphor Discourse Analysis (CMDA). Specifically, I included an expanded discussion of CDA’s theoretical principles and its applicability to analyzing metaphorical discourse in media contexts, grounding this discussion in established scholarship (e.g., Charteris-Black, 2004; van Dijk, 1997).

**b. Integration of Suggested References:** To further enhance theoretical depth, I have incorporated the references Review #1 provided: Lu & Yu (2024), addressing metaphorical discourse in COVID-19 vaccine narratives within Chinese and American media. Yu et al. (2024), focusing on corpus-assisted critical discourse analysis of media representation, illustrating practical applications of CDA. Lu et al. (2022), highlighting image construction through media discourse analysis.

These citations have been strategically positioned to strengthen the theoretical framing in the literature review and directly referenced in the discussion section, reinforcing the coherence and continuity between theoretical foundations and empirical findings.

**c. Strengthened Linkages in the Discussion Section**:

The Discussion section has been revised to explicitly reference the relevant literature review earlier, thus better aligning empirical observations with theoretical insights and existing scholarship.

For 5.1, I have added:

The findings reinforce previous research indicating that metaphors significantly influence public perceptions by embedding ideological narratives within media discourse (Dong, 2023; Liu, 2023). Consistent with Yang (2023) and Zhang and Lin’s (2023) observations that metaphors serve both persuasive and emotive functions during crises, this study demonstrates how Western media strategically use negatively charged metaphors—such as “manipulative politician” or “dishonest vendor”—to frame China’s humanitarian aid.

For 5.2, I have added:

This study further extends existing scholarship on metaphorical framings. Whereas earlier studies have primarily focused on war, architecture, journey, or body metaphors (Zhang & Lin, 2023; Yang, 2023), our analysis specifically addresses the previously under-examined use of human metaphors. By explicitly attributing human traits to nations, these metaphors carry potent ideological undertones, reflecting and reinforcing particular geopolitical narratives. It supplements Lu and Yu’s (2024) findings, the distinct metaphorical portrayals reflect deeper ideological contrasts and sociocultural contexts, highlighting Western media’s implicit geopolitical biases during the pandemic.

For 5.3, I have added:

The findings presented above explicitly illustrate the frame-building process articulated in framing theory (Goffman, 1974; Gitlin, 2003; Muñiz, 2020). As Beratšová et al. (2018) noted, media framing is influenced by structural factors such as political stance, ownership structure, and demographic variables of target audiences. Consistent with previous research highlighting the use of conflict and negativity frames (Gabore, 2020; Sun, 2021), *The Australian*’s portrayal illustrates the “China threat” narrative, driven by its conservative ideological stance and corporate ownership, and *The Guardian*’s ambivalent yet critical stance underscores how editorial independence allows nuanced framing, resonating with its progressive audience. This results support previous framing research by highlighting that media representations of China reflect diverse, strategic editorial choices shaped by complex interactions of ideological, economic, and socio-cultural factors (Lams, 2016; Rodríguez-Wangüemert, 2019; Liu et al., 2022).

Therefore, I believe these revisions substantially improve the clarity and rigor of my manuscript. Thank Review #1 again for his/her constructive comments.

**REVIEWER #2**

1.The projects funded by the stated sources are unrelated to the current paper.

**Response:** Thank Review #2 for his/her comment.I confirm that although this study received general support from the Tianjin Education Commission Research Project [Grant number 2024SK022], the funders **had no role in study design, data collection and analysis, decision to publish, or preparation of the manuscript**. Accordingly, I have revised the Funding Statement to clearly reflect this, in line with PLOS ONE’s policy. The updated Funding Statement now reads:

**"This work was supported by Tianjin Education Commission Research Project, China [Grant number 2024SK022]. The funders had no role in study design, data collection and analysis, decision to publish, or preparation of the manuscript."**

I hope this clarification is acceptable.

2. The research methodology and results are not clear. The search period is stated to end on June 1, 2024, but Figure 1 in the results section shows data up to December 2024. Additionally, the sub-corpus of Western English-speaking News Reports on China’s Humanitarian aidt to Europe contains 21 valid samples, yet Section 4.3 mentions 23 articles related to "mask diplomacy."

**Response**: Thank for Review #2 for his/her careful review and for pointing out the inconsistencies in my methodology and results. I appreciate his/her detailed observations. In response, I have made the following revisions:

**a. Search Period Discrepancy:** I acknowledge the concern regarding the discrepancy between the stated search period and the timeline displayed in Figure 1. To clarify, the data collection was strictly conducted with a search period ending on June 1, 2024, as indicated in the methodology. The discrepancy results from Factiva’s default visualization settings, which automatically present frequency data across standardized annual intervals, causing the x-axis to extend beyond the actual retrieval period. Unfortunately, this visualization format cannot be manually adjusted in Factiva.

To address this and avoid confusion, I have added a **clarifying note in the figure caption** to explicitly state the actual search period and explain the technical reason for the extended timeline. The underlying data remain accurate and fully consistent with the study design.

**b.Sub-corpus Sample Count Inconsistency:** I have reviewed my data and confirm that the final sub-corpus comprises 21 valid articles. The mention of 23 articles in Section 4.3 was an oversight. I have updated Section 4.3 to consistently report the correct number of valid samples as 21.

I believe these revisions clarify my methodology and results. Thanks again for his/her valuable feedback, which has helped me improve the transparency and accuracy of my manuscript.

3. The corpus used for Critical Metaphor Discourse Analysis (Sub-corpus) consists of only 21 samples, which is likely too small to draw conclusions with broad generalizability.

**Response:** Thank Review #2 for his/her comment regarding the size of my corpus for CEMAE. I acknowledge that a corpus of 21 samples may appear limited for drawing conclusions with broad generalizability. However, I would like to emphasize that my study is primarily qualitative in nature. CMDA focuses on an in-depth analysis of linguistic and cognitive features rather than statistical generalizability. The selected 21 articles were carefully curated from reputable Western news sources to capture the specific discourse surrounding China’s Humanitarian aidto Europe during a distinct event. Their rich, focused content provides meaningful insights into the construction of metaphors and the associated cognitive interpretations.

In response to this comment, I have added a discussion of this limitation in the manuscript’s Limitations section. I also clarify that while my findings are based on a limited corpus, they offer valuable, in-depth qualitative insights. Future research with a larger dataset may further validate and expand on my results. I hope these clarifications adequately address your concern.

4. The study sample is limited to four Western media outlets, treating "Western media" as a monolithic group and overlooking the potential variations and nuances between different media organizations. The political stance, ownership structure, and target audience of individual media outlets can significantly influence the portrayal of China's image. It is recommended to differentiate between various types of media.

**Response:** Thank Review #2 for his/her valuable feedback regarding the selection of my study sample. I do agree that treating “Western media” as a homogeneous group may overlook important variations. It is admitted that I have already mentioned in the Limitations section to acknowledge that the sample of four mainstream Western media outlets may not fully capture the diverse range of perspectives across different types of media. I recognize that individual media organizations can vary significantly. Also, in the Methods section, I have clarified that the selection of these four reputable outlets was intentional, based on their prominence and influence in global media discourse. I also note that my study is exploratory and that future research should extend the analysis to include a broader range of media sources to further validate and expand upon my findings. However, I am fully aware that these clarifications are not sufficient enough to satisfy this topic.

Although my current dataset is limited to four outlets, I have revised the Results and Discussion sections to include differentiated observations for each outlet (i.e., The New York Times, The Guardian, Der Spiegel, and The Australian). I now discuss how factors such as political stance and target audience might contribute to the variations in their portrayal of China’s emergency humanitarian aid. The following is my revised Discussion part:

**5.3 Media Outlet’s Positionality and Its Framing of Nation’s Image**

Notably, Western media outlets exhibit nuanced variations in their framing of the conceptual metaphor PERSON, reflecting their diverse positions on China’s image.  The portrayal of China’s image in international media is often shaped by a complex interplay of factors, including the political stance, ownership structure, and target audience of individual media outlets. Each of these elements can influence how China is represented, reflecting broader ideological, economic, and cultural dynamics. These factors shape the narratives and metaphors employed, such as CHINA AS POLITICIAN, resulting in diverse representations across different publications.

For the political stance, *The New York Times* is generally perceived as having a center-left bias, often endorsing liberal policies and Democratic candidates. It is owned by The New York Times Company, with the Ochs-Sulzberger family maintaining control through a dual-class share structure that ensures editorial independence. It aims to cater to an urban, educated readership, with a significant portion interested in in-depth political and international coverage.  In its coverage, it employs a nuanced approach, using sarcasm to critique European countries’ “flattering” attitudes toward China, suggesting such praise compromises their dignity. Employing dictions of “rewarding friends” and “punishing perceived enemies” characterize China’s political strategies, blending irony with a modicum of respect, which reflects the *The New York Times*’s commitment to critical analysis, in line with its liberal perspective and intellectually engaged audience. In contrast, *The Guardian*, is known for its left-leaning orientation, advocating progressive and liberal viewpoints. Owned by the Guardian Media Group, which is in turn owned by the Scott Trust, *The Guardian* operates under a unique structure that ensures editorial independence and freedom from commercial pressures. Its readership is diverse, sharing a passion for insightful journalism, progressive values, and thought-provoking content. In framing CHINA AS POLITICIAN, *The Guardian* narrates China as a culpable politician responsible for fostering mistrust within the EU, using expressions such as “unfortunately, the mask diplomacy works” to convey regret and distress. Meanwhile, *The Australian* is known for its conservative orientation, often supporting right-leaning policies and perspectives.  In framing CHINA AS POLITICIAN, *The Australian* adopts an overtly derogatory tone, using phrases like “ill-conceived attempts” and “a propaganda win” to directly map China as a manipulative politician, describing its actions as “a fiasco.” This framing reflects its conservative stance and resonates with readers skeptical of China’s global intentions.Thus, while the conceptual metaphor CHINA AS POLITICIAN is employed across Western media, each outlet’s portrayal varies according to its ideological stance and intended audience, with American media distancing themselves by positioning Europe as a victim, European media expressing ambivalence, and Australian media demonstrating strong opposition to China’s actions. Therefore, the political alignment of a media outlet plays a crucial role in shaping its coverage of China. Media organizations with distinct ideological leanings—whether liberal, conservative, or neutral—are likely to interpret and present information about China in ways catering to their political audiences. As media organizations continue to navigate complex geopolitical and economic realities, the portrayal of China will remain subject to the intricate dynamics of political ideology, ownership interests, and audience expectations.

The findings presented above explicitly illustrate the frame-building process articulated in framing theory [12][11][30].  Consistent with previous research highlighting the use of conflict and negativity frames [10][37], *The Australian*’s portrayal illustrates the “China threat” narrative, driven by its conservative ideological stance and corporate ownership, and *The Guardian*’s ambivalent yet critical stance underscores how editorial independence allows nuanced framing, resonating with its progressive audience. This results support previous framing research by highlighting that media representations of China reflect diverse, strategic editorial choices shaped by complex interactions of ideological, economic, and socio-cultural factors [21] [25][35] .

I believe my clarifications and these revisions address his/her concerns by providing a more nuanced discussion of the media landscape and clearly clarifying the rationale behind my sample selection.

5. The study lacks an interpretation of the cultural and historical context. For instance, while a statistical analysis of the frequency of "mask diplomacy" is provided, the underlying meanings of the data and their connection to Western political and cultural narratives are not sufficiently explored. This is also evident in the analysis of China's dual image in Western media.

**Response:** Thank Review #2 for his/her insightful feedback regarding the need to further interpret the cultural and historical context of my findings. I agree that understanding the underlying meanings of the statistical data on “mask diplomacy” and the analysis of China’s dual image requires a deeper exploration of the Western political and cultural narratives. In response, I have revised the manuscript in the following ways:

**a.In the Literature Review**, I have added a focused part about how “mask diplomacy” emerged within specific geopolitical contexts, and how its usage reflects long-standing Western narratives of skepticism, containment, and the “Othering” of China. The following is my revised Discussion part:

**2.3. Mask diplomacy**

The concept of “mask diplomacy” emerged during the COVID-19 pandemic. It was first proposed by Wong (2020) in *The Diplomat*, with his analysis of three features, particularly in relation to China’s efforts to improve its global image and expand influence, with skepticism toward China’s humanitarian aid [42]. This terminology reflects broader historical and geopolitical narratives prevalent in Western discourse, notably the portrayal of China as a strategic and potentially threatening “Other”. Then the concept has also been discussed in studies related with China’s humanitarian aid to countries in Europe and the Middle East and North Africa (MENA) region as part of its Belt and Road Initiative [9] [18]. One study indicates that China’s mask diplomacy efforts significantly improved media tone and increased the reproduction of China’s preferred narratives in recipient countries [30].

However, the majority of the existing scholarship has noted that western perceptions of China’s global engagement are often characterized by suspicion, containment, and critical evaluations of China’s intentions and actions [17] [30]. Thus, the widespread adoption of “mask diplomacy” rather than more neutral terms like “humanitarian aid” or “medical assistance” signals an ideological framing that aligns with historical Cold War narratives, ongoing containment policies, and neo-Orientalist perspectives. Despite growing academic attention to geopolitical dimensions of mask diplomacy, few studies explicitly interrogate how this term functions discursively within Western media narratives. This gap highlights the necessity of critically analyzing the ideological underpinnings and cognitive implications embedded in the metaphorical use of “mask diplomacy”, a core focus of the present study.

**b.Enhanced Cultural and Historical Context:** I have added a dedicated subsection in the Discussion section that interprets our findings within the broader cultural and historical context. This new section elaborates on how historical perceptions of China and prevailing Western political narratives (such as the legacy of Cold War tensions and Eurocentric perspectives) influence the interpretation of “mask diplomacy.” I have supplemented it to the Discussion section. The following is my revised Discussion part:

These metaphorical framings resonate deeply with broader Western ideological constructs and historical narratives. Depictions such as China as “an authoritarian dictator” or “an ambitious aggressor” reflect enduring Cold War mentalities, highlighting Western anxieties toward non-democratic powers. Similarly, the portrayal of China as “a dishonest vendor” draws upon historically entrenched, neo-Orientalist stereotypes that emphasize economic suspicion and moral inferiority. Such language reinforces an “us-versus-them” binary inherent in Orientalist discourse, legitimizing geopolitical containment and skepticism toward China's global initiatives. Thus, Western media’s strategic metaphorical framing during the pandemic illustrates how language simultaneously invokes and perpetuates deep-seated ideological and cultural narratives [18][24][33].

While existing studies have addressed the geopolitical implications of China’s humanitarian actions during the COVID-19 pandemic from the perspective of international relations and soft power diplomacy [33], this study contributes a complementary lens by offering a discourse-level, metaphor-focused media analysis. By tracing how different Western media outlets metaphorically construct China as various kinds of human agentsthis research reveals the cognitive strategies and ideological functions underlying seemingly neutral humanitarian narratives. Such metaphorical framings not only shape public perception but also reflect broader anxieties regarding China’s rising global status and contested image in Western discourse.

I believe these revisions substantially enhance the manuscript by providing a richer interpretative framework that connects our empirical findings to the broader cultural and historical narratives. Thank you again for your constructive suggestions.

6. "Mask diplomacy" is a key concept in the paper; however, it lacks an in-depth exploration of its multifaceted political implications.

**Response:** Thank Reviewer #2 for the thoughtful suggestion regarding the need to further explore the political implications of “mask diplomacy.” I greatly appreciate this perspective and fully recognize its relevance.

However, as this study is grounded in Critical Discourse Analysis, its primary aim is not to investigate China’s geopolitical motivations or the strategic outcomes of mask diplomacy, but rather to examine how Western media discursively construct the meaning of “mask diplomacy” through consistent lexical choices and metaphorical framing. Specifically, the research focuses on how the term “mask diplomacy” — rather than more neutral terms like “humanitarian aid” — is employed across different outlets to build ideological narratives about China during the COVID-19 pandemic.

The analysis reveals that although the event reported is the same — China’s humanitarian aid to Europe — different media outlets apply metaphorical framings under the conceptual metaphor THE NATION AS PERSON to construct divergent representations of China (e.g., as “a manipulative politician,” “a dishonest vendor,” or “an ambitious aggressor”). These framings are shaped by each outlet’s political stance, ownership structure, and target audience, and they influence public perception by embedding ideologically charged meanings into seemingly descriptive narratives.

That said, I fully agree that the term “mask diplomacy” carries broader political connotations. Therefore, I have expanded the **literature review** to include an overview of existing scholarship on the concept and its global application. I also added a paragraph in the **discussion section** to briefly acknowledge the political significance of humanitarian aid as a soft power tool. These revisions aim to better contextualize the discourse analysis without shifting the focus away from the core objective of the study.

Still, I fully recognize the value of his/her suggestion. In response, I have made the following additional revisions to the manuscript:

**a.In the Literature Review**, I have added a section that traces the origins and definitions of “mask diplomacy,” including its use in global health diplomacy and China’s soft power strategy. The following is the added part in Literature Review.

The concept of “mask diplomacy” emerged during the COVID-19 pandemic, firstly proposed by Wong(2020) in the website of The Diplomat (<https://thediplomat.com>), with his analysis of three features, particularly in relation to China’s efforts to improve its global image and expand influence. Then the concept has also been discussed in studies related with China’s humanitarian aid to countries in Europe and the Middle East and North Africa (MENA) region as part of its Belt and Road Initiative (Kowalski, 2021; Fahrisa et al., 2023). This strategy aimed to counter negative perceptions stemming from the virus’s origin in Wuhan and showcase China as a benevolent global leader (Kowalski, 2021; Müller et al., 2024). Studies indicate that China’s mask diplomacy efforts significantly improved media tone and increased the reproduction of China's preferred narratives in recipient countries (Müller et al., 2024). Despite the strategic significance and international implications of mask diplomacy, existing scholarly research specifically addressing China’s image within the context of mask diplomacy remains limited, with fewer than five studies directly focusing on this topic. This notable scarcity underscores the necessity and value of further research in this area, highlighting the originality and relevance of the present study.

**b. In the Discussion section,** I have revised specifically within Section 5.2 (Ideological Implications of THE NATION AS PERSON), to briefly address the symbolic power of “mask diplomacy” in international relations. The newly added paragraph discusses how the use of this term in Western media reflects not only linguistic preference, but also ideological positioning in the context of global power dynamics. This addition situates the metaphorical framing of China’s humanitarian aid within broader political and discursive tensions, without shifting the primary focus of the study away from discourse analysis.

This paragraph serves as a transitional section that bridges the macro-political implications of “mask diplomacy” and the micro-level discourse strategies analyzed in the paper. It both responds directly to the reviewer’s comment and strengthens the coherence of the paper by linking geopolitical context to media language choices. The following is the added paragraph in Section 5.2:

The primary aim of this study is to examine how Western media discursively construct the notion of “mask diplomacy,” yet it is also important to acknowledge the political significance embedded in the term. The consistent use of “mask diplomacy” instead of more neutral phrases like “humanitarian aid” reflects not only lexical preference, but also ideological positioning. In global discourse, humanitarian aid is increasingly perceived as an instrument of soft power and symbolic diplomacy. As previous studies have shown (Kowalski, 2021; Müller et al., 2024), China’s pandemic-time medical outreach was widely interpreted as an effort to rehabilitate its international image and project itself as a benevolent global leader. Western media’s metaphorical framing of this initiative, therefore, may not be ideologically neutral, but rather a reflection of deeper geopolitical anxieties and skepticism surrounding China’s global rise.

**c. In the Limitations section**, I have added a reflection on the need for future studies to investigate China’s humanitarian aid more explicitly as a form of diplomatic practice, especially from a political science or international relations perspective. The following is the added part in Limitations.

Finally, while this study focuses on media framing, it does not explore China’s humanitarian aid as a diplomatic strategy. Future studies may examine how humanitarian assistance functions as a tool of soft power and how it is received in different geopolitical contexts.

**d. Additional References:** To support this enhanced discussion, I have incorporated relevant citations from recent literature that analyze the political implications of mask diplomacy. These references provide empirical and theoretical backing for our arguments. These are the references I have added:

Omelicheva M, Carter B. COVID-19 “Humanitarianism”: Geopolitical logics of Chinese, American, and Russian assistance. Geopolitics and International Relations Series. Vol. 4. 2024.

Lu G, Chen J, Zhang L. The image construction of the City Yiwu in overseas media: Corpus-driven research based on 2010–2015 and 2016–2021. Linguistics and Culture Review. 2022;6(1):162–176.

Lu G, Yu Y.The use of metaphors to construct crisis discourses in describing COVID-19 vaccines in the Chinese and the American news media: A corpus-assisted critical approach. *Journal of Language and Politics.* 2024;23(4):520–543.

Liu M, Huang J. Framing responsibilities for climate change in Chinese and American newspapers: A corpus-assisted discourse study. Journalism. 2024;25(8):1792–1811.

Beyond the itemized responses to Reviewer #1, Reviewer #2, and the Academic Editor, I have also reviewed and responded to the comments included in the attached feedback document. The following section presents my point-by-point replies to those additional remarks.

1. Please maintain consistency in terminology usage, particularly regarding capitalization - e.g., covid-19, COVID-19, and China's Medical Assistance. When a convention is established, it should be maintained throughout the manuscript.

**Response：**Thank Reviewer for his/her careful attention to detail. I have thoroughly reviewed the manuscript to ensure consistency in terminology and capitalization. All instances of “covid-19” have been standardized to “COVID-19,” and consistent formatting has been applied to terms such as “China’s medical assistance.” I appreciate your helpful reminder, which has improved the clarity and professionalism of the manuscript.

2. Please review and ensure full compliance with APA formatting guidelines.

**Response：**Thank reviewer for his/her comment regarding formatting consistency and compliance with APA style. I would like to clarify that PLOS ONE does not follow APA referencing guidelines. Instead, the journal adopts a modified **Vancouver style** for all in-text citations and reference formatting, as specified in the PLOS ONE submission guidelines. Accordingly, I have revised the entire reference list and in-text citations to conform fully to the **PLOS ONE style**.

3. The connection between the literature review and your research findings requires strengthening (notably, the discussion section lacks citations). Specifically, the rationale for employing Critical Discourse Analysis (CDA) needs clarification. If CDA is being utilized as a theoretical framework, it should be thoroughly introduced in the literature review, potentially in section 2.2. Consider incorporating the following references to enhance theoretical depth:
a. Lu, G., & Yu, Y. (2024). The use of metaphors to construct crisis discourses in describing COVID-19 vaccines in the Chinese and the American news media: A corpus-assisted critical approach. *Journal of Language and Politics*, 23(4), 520-543.
b. Yu, Y., Lu, G., Fung Sin, K., & Yu, Z. (2024). Media representations of China’s inclusive education: A corpus-assisted critical discourse analysis. *PLoS ONE*, 19(3), e0300123.
c. Lu, G., Chen, J., & Zhang, L. (2022). The image construction of the City Yiwu in overseas media: Corpus-driven research based on 2010-2015 and 2016-2021. *Linguistics and Culture Review*, 6(1), 162-176.

**Response：**

I have already Strengthened the Connection Between Literature Review and Findings. Furthermore, the justification for Employing Critical Discourse Analysis (CDA) is also supplemented. Integration of Suggested References. Last, I have incorporated the suggested references to enhance the theoretical depth of our study: **Lu & Yu (2024)**: This study’s corpus-assisted critical approach to metaphor analysis has been referenced to support our methodology, particularly in analyzing how metaphors shape crisis discourses. **Yu et al. (2024)**: We have cited this paper to reinforce our discussion on how media representations construct social narratives, drawing parallels to the framing of China’s humanitarian aid. **Lu, Chen, & Zhang (2022)**: This research on city image construction has been referenced in our literature review to contextualize how China’s international portrayal has evolved over time in foreign media.

All the detailed revision **has been mentioned** in the previous response to Reviewer #2.

4. The data selection methodology requires restructuring. Several points need addressing:
a. Please justify why secondary searches using "China's emergency medical" were conducted in WENRCC rather than directly in Factiva

**Response：**Thank Reviewer for his/her thoughtful question regarding the search procedure.

The reason for conducting secondary keyword searches (e.g., “China’s emergency medical assistance”) within the WENRCC **corpus**( later revised into CC Corpus in my revised version), rather than directly in Factiva, lies in both **methodological consistency** and **research precision**: The WENRCC corpus was initially built using broad yet targeted search terms (“COVID-19,” “Coronavirus,” and “China”) to capture a comprehensive dataset focused specifically on how **China’s role in the pandemic** was framed in Western media. This ensured that all texts within the corpus had already met basic thematic relevance to the study. Also, by limiting secondary searches (e.g., “medical assistance,” “humanitarian aid,” “mask diplomacy”) to within the pre-filtered WENRCC, I could ensure that the results were **discursively situated within COVID-19-related narratives about China**—rather than returning unrelated or loosely connected global humanitarian aid articles that might arise from a full Factiva search. Finally, the study adopts a **corpus-assisted critical discourse analysis** approach, which benefits from working within a structured and thematically unified textual environment (Corpus CC). Searching within this corpus rather than the entire Factiva database enhances internal consistency and analytic focus.

This two-stage search method—first building a relevant super-corpus, then refining with topic-specific terms—is also consistent with existing methodologies in critical metaphor and discourse studies.

I hope this clarifies the rationale behind this research design decision. Thank you again for the opportunity to elaborate on this point.

b. The corpus size (22 articles) appears insufficient for robust analysis (specific token counts needed)

**Response：**I have already mentioned my revision concerning this part in the previous Reviewer #2 response 3.

c. The frequency tables showing predominantly single occurrences raise reliability concerns

**Response：**Thank reviewer for his/her comment regarding the frequency tables and the predominance of single occurrences. While I acknowledge that many metaphorical expressions identified in the corpus appear only once, I would like to emphasize that the aim of this study is **not statistical generalization**, but rather **qualitative interpretation through Critical Metaphor Discourse Analysis (CMDA)**. As noted by Charteris-Black (2004), even metaphors that occur infrequently can carry significant ideological weight within media discourse, especially when they are **strategically positioned** or embedded in **evaluative and framing structures**.

Moreover, the relatively small size of the CEMAE sub-corpus (n = 21) reflects the narrow focus of the study on a highly specific discourse event—Western media framing of China’s humanitarian assistance to Europe during early COVID-19. In this context, the analytical emphasis lies in **identifying conceptual metaphors** and **interpreting their ideological implications**, rather than establishing frequency-based trends. Nonetheless, to strengthen the study’s transparency, I have clearly acknowledged this limitation in the revised manuscript and clarified that the findings are intended to offer in-depth qualitative insights rather than broad generalizations.

d. CDA typically acknowledges ideological variations even within national media (broadsheets, tabloids, left-wing, right-wing outlets). The rationale for aggregating news coverage from different countries requires further justification, particularly regarding shared ideological perspectives

**Response：**Thank reivewer for this insightful observation. I fully agree that Critical Discourse Analysis (CDA) emphasizes the importance of ideological variation not only across national contexts but also within media systems (e.g., broadsheet vs. tabloid, left- vs. right-leaning outlets). This principle is particularly relevant when examining how national images are constructed and contested. In this study, I aggregated data from four leading Western English-language newspapers across the U.S., U.K., Germany, and Australia, not to imply a uniform ideological position, but to examine whether and how a **shared discursive metaphor**—namely, “mask diplomacy”—was employed transnationally to frame China’s medical assistance. The focus is therefore not on comparing national ideological stances per se, but on **tracing common metaphorical patterns** across culturally and politically diverse media systems. This approach aligns with CDA’s attention to both **local variation** and **global discourse flows** (van Dijk, 1997; Wodak & Meyer, 2009).

To address potential ideological heterogeneity, I have added a new section (5.3) discussing each outlet’s **political orientation, ownership structure, and audience expectations**, showing how these factors mediate the metaphorical framing of China. This addition provides a more nuanced analysis of intra- and inter-national variation and supports a critical interpretation of the “mask diplomacy” metaphor as a transnational but ideologically inflected discourse strategy.

5. Sections 4.1-4.2 could benefit from implementing a keyword list-driven framing theory approach. Consider referencing:
a. Ming, L., & Ma, J. (2022). The politics of fear in Hong Kong protest representations: A corpus-assisted discourse study. *Journal of Language and Politics*, 21(1), 37-59.
b. Liu, M., & Huang, J. (2024). Framing responsibilities for climate change in Chinese and American newspapers: A corpus-assisted discourse study. *Journalism*, 25(8), 1792-1811.

**Response：**Thank reviewer for the valuable suggestion regarding the application of a keyword list-driven framing theory approach in Sections 4.1 and 4.2. I appreciate the recommended references by Ming & Ma (2022) and Liu & Huang (2024), both of which offer insightful models for corpus-assisted frame analysis. In the current study, I adopt **Critical Metaphor Discourse Analysis (CMDA)** as the core methodology, with framing theory primarily used as a **complementary interpretive lens** in the discussion (Section 5.3), particularly in relation to how metaphors function as framing devices. While keyword-driven frame identification is highly effective for analyzing large-scale textual corpora, my sub-corpus comprises only 23 texts. Given this small and qualitatively curated dataset, a metaphor-centered close reading was deemed more appropriate for uncovering **latent ideological framing** and **semantic associations** (Charteris-Black, 2004).

Nonetheless, I agree that framing theory enriches the theoretical depth of the analysis. In response to this comment, I have strengthened the integration of framing theory in the discussion section, especially by elaborating on how metaphorical patterns (e.g., “China as politician” or “Europe as victim”) in line with **issue-specific frames** such as **responsibility**, **morality**, and **threat**.

6. The Discussion section requires substantial revision to address the aforementioned concerns.

**Response：I have already mentioned this revision in** concerning this part in the previous Reviewer #1 response 3; Reviewer #2 response 4.

Thank you very much again for these meticulous suggestions. I do benefit a lot and revised mostly to resubmit my paper.

Sincerely,

Lei Sun

Tianjin Normal University
