# Supplementary material for: On China’s image constructed from western news coverage of China’s humanitarian aid
Source: PLoS One. 2025 Jun 18;20(6):e0326214. doi: 10.1371/journal.pone.0326214 (PMC12176173; doi:10.1371/journal.pone.0326214)
Supplement: Attachment — Submitted filename: comments_R1.docx [file pone.0326214.s007.docx]

1. Methodology section
a. I noticed that in your manuscript, Section 3.2 is titled “Methodology.” However, “methodology” generally refers to a broader framework that encompasses the overall approach, including data, tools, and analysis. In Section 3.2, you only describe the specific method used in this project. Therefore, I recommend renaming Section 3 as “Methodology” and Section 3.2 as “Method.”
b. Additionally, the second paragraph of Section 3.1.1 contains extensive explanations about the criteria for selecting the newspapers. To enhance clarity and conciseness, you might consider presenting that information in a table.
c. I also suggest combining Sections 3.1.1 (Data Collection) and 3.1.2 (Corpus Construction) since they are closely related and share overlapping content.
d. Moreover, some details within the current methodology section appear to be better suited for the “Findings” section. Please consider relocating this content accordingly.
e. Finally, the manuscript would benefit from a dedicated data analysis section that demonstrates, for example, showing your readers an example about how metaphor(s) is identified in the dataset (i.e., MIP or MIPVU).
f. Based on these points, I recommend restructuring the methodology section as follows:

3. Methodology

3.1 Data

3.2 Methods

3.3 Data Analysis

I hope these suggestions help improve the clarity and structure of your manuscript.

2. Terminology issues:
a. AntConc

In the quantitative analysis section, you refer to AntConc 3.5.9 as **a vocabulary analysis software**. However, AntConc is more described as a “corpus analysis toolkit” (please also cite from Anthony). I recommend updating the description to reflect its true functionality.

b. MIP vs. MIPVU

In Section 3.2, the manuscript states that the MIP method was utilized in this project, while in Section 4.4, a different method, MIPVU, is mentioned. Please ensure consistency in the naming of the method throughout the manuscript and cite the related reference for the chosen method.

c. Section 4 (Results)

Please note the essential difference between “Results” and “Findings”:

The former one should present the empirically obtained data, including statistical analyses, charts, and other measurable experimental indicators, whereas the later one is intended for interpreting, summarizing, and analyzing these data.
